# Effects of insertion torque values on the marginal bone loss of dental implants installed in sheep mandibles

**DOI:** 10.1038/s41598-021-04313-5

**Published:** 2022-01-11

**Authors:** Sergio Alexandre Gehrke, Jaime Aramburú Júnior, Tiago Luis Eirles Treichel, Tales Dias do Prado, Berenice Anina Dedavid, Piedad N. de Aza

**Affiliations:** 1 Department of Biotechnology, Universidad Católica de Murcia, 30107 Murcia, Spain; 2 Department of Research, Biotecnos - Technology and Science, Cuareim 1483, 11100 Montevideo, Uruguay; 3 Department of Surgery, Faculty of Medicine Veterinary, University of Rio Verde, Rio Verde, Brazil; 4 Department of Materials Engineering, Pontificia Universidade Católica do Rio Grande do Sul, Porto Alegre, 90619-900 Brazil; 5 Department of Materials, Instituto de Bioingenieria, Universidad Miguel Hernández, Elche, Alicante Spain

**Keywords:** Biological techniques, Biotechnology, Medical research, Engineering, Materials science

## Abstract

The aim of the present in vivo study was to analyze and compare the effects on the crestal bone healing of two different implant macrogeometries installed in fresh socket areas and in normal bone areas with different insertion torque values. Two implant macrogeometries were used in the present study, DuoCone implant (DC) and Maestro implant (MAE), forming four groups: group DCws, in which the implants were installed in healing bone (without a socket); group DCfs, in which the implants were installed in post-extraction areas (fresh sockets); group MAEws, in which the implants were installed in healing bone (without a socket); group MAEfs, in which the implants were installed in post-extraction areas (fresh sockets). After 30 and 90 days of implantations in the bilateral mandibles of 10 sheep, eighty implants were evaluated through digital X-ray images and histologic slices. The crestal bone position in relation to the implant platform shoulder was measured and compared. The measured insertion torque was 47.2 ± 4.69 Ncm for the DCws group, 43.4 ± 4.87 Ncm for the DCfs group, 29.3 ± 3.16 Ncm for the MAEws group, and 27.7 ± 4.41 Ncm for the MAEfs group. The radiographic mesio-distal and histological bucco-lingual analyses showed significantly greater vertical bone loss in the implants installed with high torque (DC groups) in comparison to the implants installed with a low torque (MAE groups) (p < 0.05), at both evaluation times. In general, low insertion torque values (Maestro implants) showed better results of MBL when compared to implants installed with higher torque values (Duo Cone implants). Moreover, our results showed that the implants installed in the sites without sockets showed a less MBL in comparison with the implants installed in sites of fresh sockets.

## Introduction

It has been shown that tooth extraction will lead to dimensional changes in the alveolar ridge together with apicocoronal and vestibulolingual remodeling of the affected area^1,2^. In addition, resorption of the alveolar buccal bone table is more pronounced than the lingual/palatal bone table^1,3^. Araujo and Lindhe^1^ suggested that 100% of the vestibular bone wall is fascicular bone that loses its function after tooth extraction and, consequently, is reabsorbed. The dimensional changes in the alveolar ridge that occur during the healing of soft and hard tissues show the greatest alterations in the first months. Subsequently, minor decreases in the ridge continue to be observed over a prolonged period^4^.

The healing process of an extraction socket consists of a series of events that include the formation of a blood clot, which is replaced by fibroreticular bone (woven bone), while the alveolar walls are gradually resorbed and remodeled^2,4^. Finally, the trabecular bone fills the extraction areas, forming a residual bone crest that continues to be remodeled throughout the patient's edentulous life^5,6^. In a study of 123 edentulous bone samples, Pietrovsky et al.^7^ demonstrated that the resorption pattern after tooth extraction is highly dependent on the alveolar process. In this sense, some authors have suggested that the placement of implants in areas of recent extraction could preserve the dimension of the alveolar ridge^8,9^. However, findings in human and dog studies could not corroborate this hypothesis and showed considerable hard tissue reabsorption after tooth extraction and immediate implant placement^10–12^. A study in humans demonstrated a narrowing of the width of the alveolar ridge around implants placed in the fresh sockets with approximately 4 mm of horizontal bone resorption^13^. In addition, the same authors demonstrated inconsistent and moderate vertical bone resorption around submerged implants placed in the recent extraction areas, with a mean value on the vestibular sides of 0.8 mm^14^.

On the other hand, one of the main and persistent concepts of implant placement is the need for adequate initial implant stability as a fundamental requirement for achieving osseointegration. Several studies have shown that implant macrogeometry has factors correlated with primary stability, such as, the body shape (cylindrical, semi tapered or tapered), cervical implant design, threads size design and pitch, apex morphology, among others^15–21^. Surgically, the installation of implants with high insertion torque values has always been accepted as a precept to ensure osseointegration. However, depending on the bone density at the site, these high torque levels can cause an increase in the inflammatory response and, in some cases, even necrosis in areas around the implant. Furthermore, Scarano et al.^22^ showed that the elevated insertion torque values produce pain and resorption of the crestal bone around the implants. Taking these concepts into account, some authors have proposed changes between the osteotomy dimension and the implant diameter, that is, a drill size that is closer to the external diameter of the implant threads, thus decreasing the insertion torque and, consequently, the compression of the bone tissue around the implant, avoiding undesirable bone effects^23,24^. Recent studies have shown that the implant placement torque can influence the response of peri-implant tissues, not only with regard to the levels of osseointegration but also the pattern of remodeling of the marginal crestal bone^25,26^.

Moreover, regarding the effects of the insertion torque in the osseointegration, some changes have been recently proposed in the macrogeometry (design) of implants, seeking to enable the reduction of insertion torque values without affecting the initial stability of the implants. In this sense, Gehrke and collaborators studied a new implant macrogeometry that has healing chambers in its body, which has been shown to reduce bone tissue compression without the need to change the sequence of burs used for osteotomy^27–29^. These studies have shown that this type of design speeds up the osseointegration time without requiring a high insertion torque value, even if the bone has a low density.

The objective of this experimental in vivo study was to evaluate the physiological bone remodeling that occurs after implant placement in areas with and without recent extraction using two implant macrogeometries, one that uses higher torques and the other that promotes lower insertion torques. The suggested hypothesis was that different implant macrogeometries can promote different effects on the marginal bone healing. A possible correlation between insertion torque (IT) and marginal bone level (MBL) was analyzed.

## Materials and methods

### Implants characteristics

**Figure 1:**
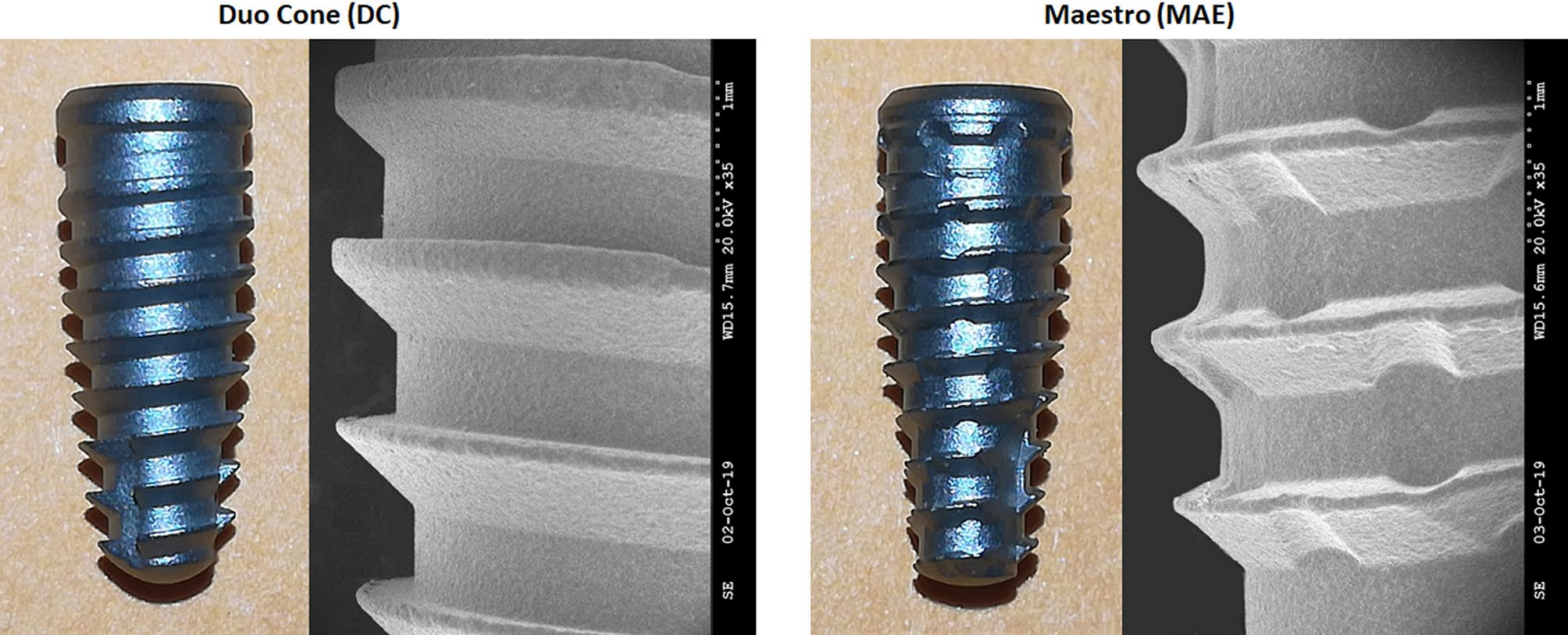
Representative image of the implant design used in the present study: DC implant with trapezoidal threads design; MAE implant with trapezoidal threads and healing chambers. SEM images with magnification of 35 times.

Eighty dental implants with Morse taper connection manufactured and marketed by Implacil De Bortoli (São Paulo, Brazil) with two distinct macrogeometries were used (n = 40 per model): Duo Cone (DC) implants, which feature trapezoidal threads and a conical design; and, Maestro (MAE) implants, which feature trapezoidal threads, conical design, and healing chambers distributed between the threads in the implant body. Both implant models used have the same type of surface treatment, that is, they are blasted with microparticles of titanium oxide (Ø100–150 µm) followed by conditioning by maleic acid. This surface presents a roughness pattern with Ra of 0.56 ± 0.10 µm^30^. Implants with the same dimensions, 4 mm in diameter and 10 mm in length, were used. In addition, each implant after insertion into the bony site received a healing abutment 3.5 mm in diameter by 3.5 mm in length (n = 80). Figure 1 shows the image of the implants and the healing abutment used in this study.

### Animal selection and care

The present study was reported in accordance with ARRIVE guidelines (https://arriveguidelines.org). Ten Santa Inês sheep of both genders from the department of surgery of the Faculty of Veterinary Medicine of the University of Rio Verde (Rio Verde, Brazil) weighing between 31 and 40 kg were used. The study was approved by the Animal Research Ethics Committee (UniRV nº07/2020) and followed the ethical principles of the National Council for the Control of Animal Experimentation (CONCEA), as well as the concern for the welfare of animals in accordance with the Law nº 11.794 of October 8, 2008 (Procedures for the Scientific Use of Animals).

Prior to surgical procedures, all animals were housed for a minimum period of 15 days to adapt to environmental conditions and human coexistence, as well as to detect possible illnesses. The animals were housed in two groups according to the time of sacrifice, that is, five animals per pen, provided with drinkers and feeders. The animals were fed industrialized chow and water ad libitum. These conditions were maintained throughout the study period.

### Anesthetic, surgical procedure and proposed groups

Due to the possibility of regurgitation and aspiration of rumen content, which can lead to asphyxia or pneumonia, the animals were fasted on solid and liquid for 24 h prior to the procedure^31^.

The anesthetic medication was a mix of atropine sulfate at a dose of 0.02 mg/kg and morphine sulfate at a dose of 0.4 mg/kg, given via intramuscular injection. After 15 min, 0.1 mg/kg of xylazine and 8 mg/kg of ketamine, both in the same syringe, were given intramuscularly. The animals were intubated (orotracheal intubation) with a flexible tube and kept on oxygen. Intravenous fluid therapy was performed with a lactated ringer solution, through cannulation of the brachycephalic vein, with a 24G catheter, throughout the surgical procedure, in a 10 to 15 ml/kg/hour venous drip. Heart rate and oxygen saturation were monitored with the aid of a pulse oximeter.

**Figure 2:**
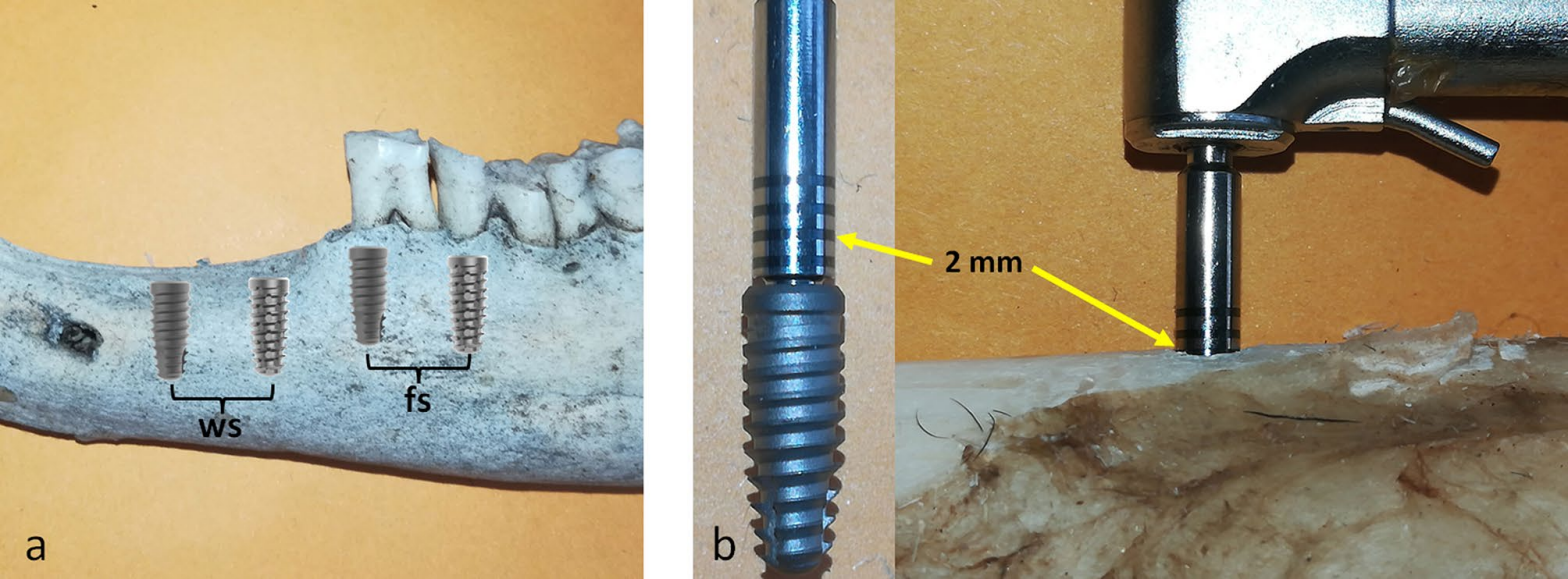
Schematic image of the predetermined positions of the implants: (**a**) two implants inserted in sites without sockets (ws) and two in post-extraction sites with fresh sockets (fs). (**b**) Image of the implant and insertion drive used to control the position of the 2 mm infra-bone, with the yellow arrows indicating the marking on the drive.

For the surgical procedure to install the implants, antisepsis of the oral cavity was performed with 0.12% chlorhexidine and local anesthetic infiltration based on 2% mepivacaine and 1:100,000 epinephrine. After 5 min of anesthetic infiltration, an incision was made over the crest of the ridge in the diastema area, passing through the gingival sulcus of the first two posterior teeth, which were carefully extracted. Thus, two osteotomies were performed in the diastema area and another two in the alveoli of the mesial roots of the extracted teeth, as shown schematically in Fig. 2a. Then, four groups were formed as follows: group DCws, in which the implants were installed in healing bone (without a socket); group DCfs, in which the implants were installed in the post-extraction area (fresh sockets); group MAEws, in which the implants were installed in healing bone (without a socket); group MAEfs, in which the implants were installed in the post-extraction area (fresh sockets). The implants were installed on both sides of the mandible in each animal, being distributed as follows: DC, MAE, DC, MAE in the left hemimandible, and MAE, DC, MAE, DC in the right hemimandible. All implants were positioned 2 mm infra-bone, which was controlled by the marking on the drive used for the implant installation, as shown in Fig. 2b.

**Figure 3:**
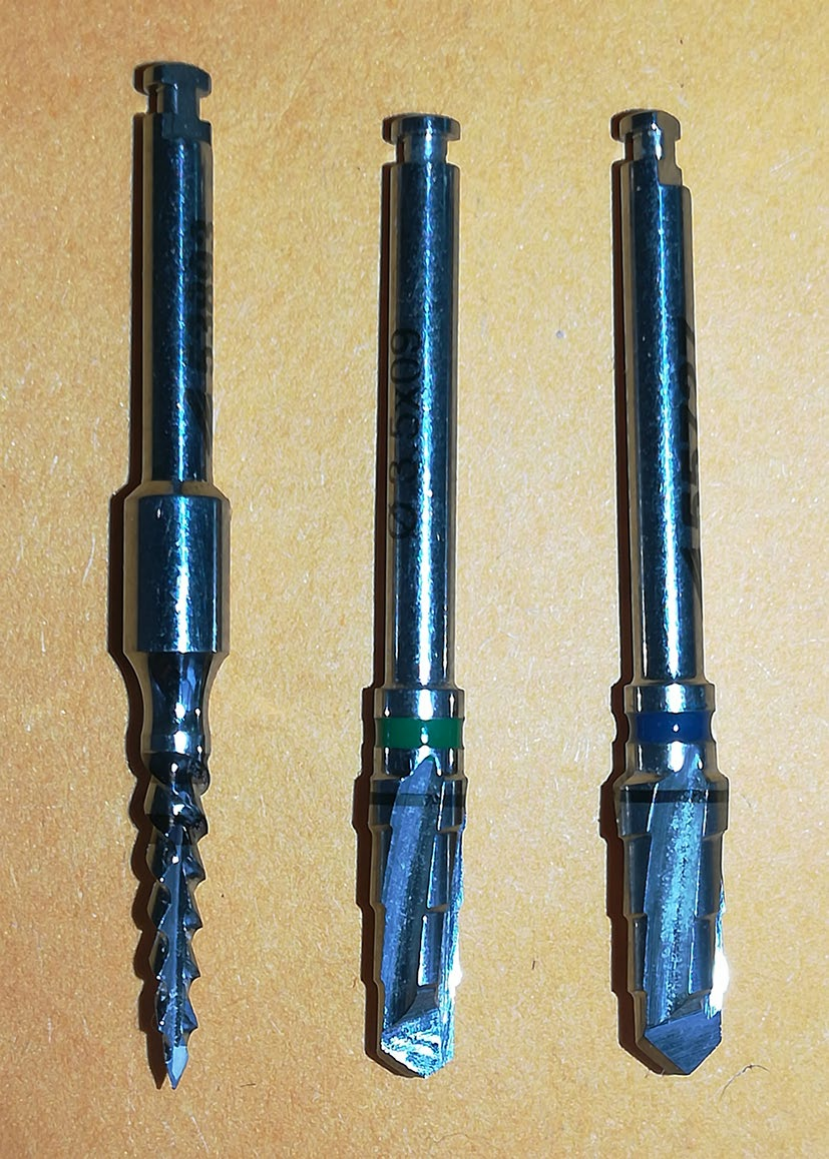
Image of the drill sequence used for all osteotomies proposed.

The osteotomies were performed with the same drill sequence (Fig. 3), according to the manufacturer's recommendations, varying the drilling speed according to the implant model: for DC implants, pilot drill 2.0 mm, 3.5 mm conical drill and 4.0 mm conical drill, all at 1200 rpm; and, for MAE implants, a pilot drill 2.0 mm at 1200 rpm, a 3.5 mm conical drill at 600 rpm and a 4.0 mm conical drill at 600 rpm. A digital surgical motor (iChiropro Bien-Air) and contra-angle 20:1 Bien-Air (Bien-Air Surgery SA, Le Noirmont, Switzerland) was used. In sites of immediate implantation in fresh sockets, these were drilled with the same sequence of drills following the path of alveoli until reaching the planned depth. All osteotomies were performed under intense irrigation with a 0.9% sodium chloride solution. All implants were installed using the motor described above (iChiropro Bien-Air) with a rotation of 30 rpm, which registered and informed, through the software coDiagnostiXTM, the maximum torque of the implant during the insertion.

After installing the 4 implants and placing the healing abutments, the mucosa was sutured with simple stitches and interrupted with 5.0 Nylon suture (Johnson & Johnson Health Care Systems Inc, Piscataway, USA). The same surgeon with extensive experience in dental implant procedures and previously calibrated performed the installation of all implants.

### Postoperative care and euthanasia

In the immediate postoperative period, the animals were monitored until full re-establishment of consciousness and then sent to their individual boxes. To control pain and inflammation, an anti-inflammatory (meloxicam 0.5 mg/kg) was administered subcutaneously, once a day for three days, and an analgesic (tramadol hydrochloride 5 mg/kg) was administered subcutaneously, three times a day for three days. In addition, a single dose of 20% oxytetracycline antibiotic (0.1 mg/kg) was administered intramuscularly. The animals were clinically evaluated once a day for physiological parameters (heart rate, respiratory rate, defecation, and urination), behavior and return to feeding.

After 30 and 90 days after surgery (n = 5 animals per time), the animals were euthanized using pentobarbital (90 mg/kg at 3%, via intravenous), according to the CONCEA guidelines for the care and use of animals for scientific and educational purposes^32^.

### Measurement of insertion torque and implant stability quotient (ISQ)

The insertion torque (IT) was measured during implant installation with the aid of the digital surgical motor, as described above, considering the maximum torque value obtained during insertion into the bone bed. The stability measured by magnetic frequency, in this case the implant stability quotient (ISQ), was performed with the aid of Osstell® (Osstell AB, Gothenburg, Sweden), and a SmatPeg sensor (type 49) was installed for each implant. For each implant, two measurements were acquired, one in the buccolingual direction and the other in the mesiodistal direction, with an overall average being made for each implant. These ISQ measurements were taken at three moments: immediately after implant placement (m1), in samples taken from animals sacrificed at 30 days (m2), and in samples taken from animals sacrificed at 90 days (m3).

### Sample preparation and histological analysis

All samples were immediately immersed in 10% buffered formalin and kept in this solution for 7 days. Then, they were dehydrated in an ethanol solution sequence (50–100%) and embedded in a historesin (Technovit 7200 VLC, Kulzer, Wehrheim, Germany). The cuts were performed using an IsoMet 1000 machine (Buehler, Lake Bluff, USA). The slides were stained using the picrosirius–hematoxylin technique^33^. Images were obtained using a Nikon E200 light microscope (Tokyo, Japan). For histological measurements, ImageJ for Windows™ software was used (National Institute of Health, Bethesda, USA). The percentage of contact between bone and implant (BIC%) was measured along the entire length of the implant surface.

### Marginal bone level (MBL) measurements

**Figure 4:**
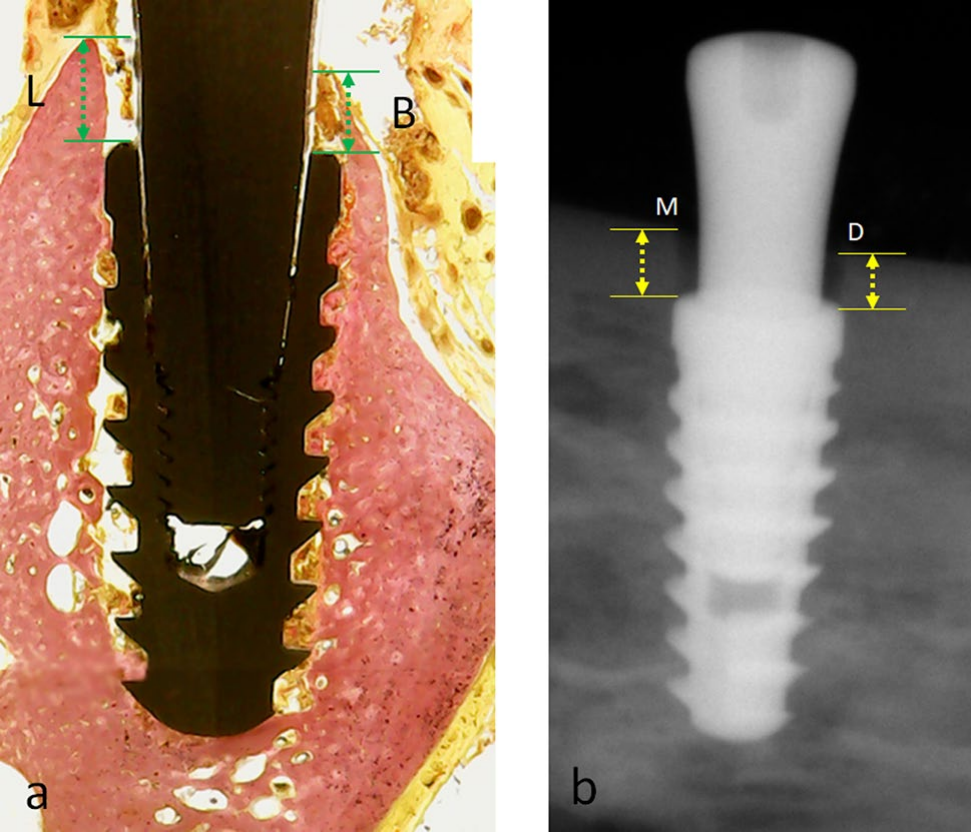
(**a**) Representative image of a histological section showing the measurements for the buccal (B) and lingual (L) marginal bone level (MBL1); (**b**) Radiographic image used to measure the mesial (M) and distal (D) marginal bone level (MBL2). All measurements were made sized from the platform shoulder to the crestal bone (MBL1 = green arrows, MBL2 = yellow arrows).

Since all implants were installed 2 mm infra-bone in relation to the implant platform, measurements were made using the histological sections for the buccal and lingual marginal bone positions (MBL1), as shown in Fig. 4a. The periapical radiographs were used to measure the mesial and distal marginal bone level (MBL2), as shown in Fig. 4b. To take the radiographic images, a digital radiography system with a portable IriX-ray DX 3000 device (Dexcowin, Seoul, Korea) was positioned at 10 cm from the sample using a digital film RVG First intraoral system (Trophy, Toulouse, France). All images were taken observing the parallelism of the equipment with the implants. The exposure time to obtain each radiograph was adjusted to 0.35 s. The images were transmitted directly to the computer using the Trophy imaging software (Toulouse, France). All measurements were taken using the ImageJ software, which was calibrated against the implant diameter as a reference.

### Sample size calculation and statistical analysis

The software SigmaStat 4.0 (Systat Software Inc, San Jose, USA) was used to calculate the sample size, based on a power of 85% to obtain a *p*-value of 0.05. Using the data (differences between the means and standard deviations), the calculated minimum sample size for each group at the 2 proposed time resulted in 8 samples. However, 10 samples were used to improve the sampling condition.

The normal distribution was tested using the D'Agostino–Pearson omnibus normality test. As the normality was confirmed, the generalized parametric linear model for repeated measures with a significance level of 5% was applied. The one-way ANOVA statistical test was used to determine the difference between the four groups in the same measured time for each parameter analyzed. The *t*-test was used to evaluate statistical differences in each parameter analyzed inside of each group among the two proposed times. The Bonferroni multiple comparison test was used to detect differences between the groups for each parameter and each time. Pearson's correlation test was used to evaluate the correlation between the IT values and BIC%, IT values and MBL1, and IT and MBL2. For all statistical tests, we used the GraphPad Prism software version 5.01 (GraphPad Software, San Diego, USA), considering the result significant when p < 0.05.

### Ethical approval

The present study was approved by the Animal Experimentation Committee (UniRV nº07/2020), University of Rio Verde (Rio Verde, Brazil). All applicable international, national, and/or institutional guidelines for the care and use of animals were followed.

### Informed consent

For this type of study, formal consent is not required.

## Results

**Figure 5:**
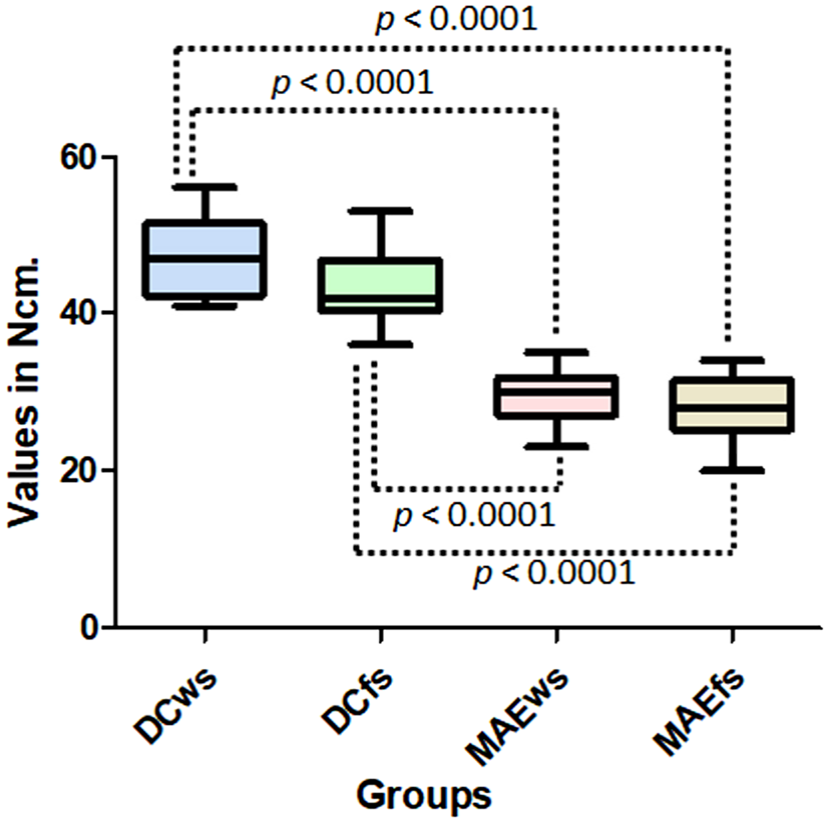
The data distribution and statistical comparison between the groups that presented differences (*t*-test) of insertion torque values.

The first parameter measured in our study was the torque during the insertion of each implant into its surgical bed, with differences in the values observed between the two implant models (ANOVA with *p* < 0.0001), regardless of the place where they were installed, with the following means and standard deviation for each group: 47.2 ± 4.69 Ncm for DCws group, 43.4 ± 4.87 Ncm for DCfs group, 29.3 ± 3.16 Ncm for MAEws group, and 27.7 ± 4.41 Ncm for MAEfs group. Figure 5 shows the data distribution with the statistical comparison between the groups that presented differences.

In clinical follow-up, no complications were observed during healing in any of the experimental areas. The peri-implant mucosa did not show signs of serious inflammation or infections outside the normal healing pattern for this type of procedure were observed in these areas during the follow-up period. Five animals were evaluated at 30 days and another five at 90 days after implant placement. Based on the clinical parameters evaluated, osseointegration was found in all the implants, since all implant areas had the healing abutments exposed at the established moments of the study.

**Figure 6:**
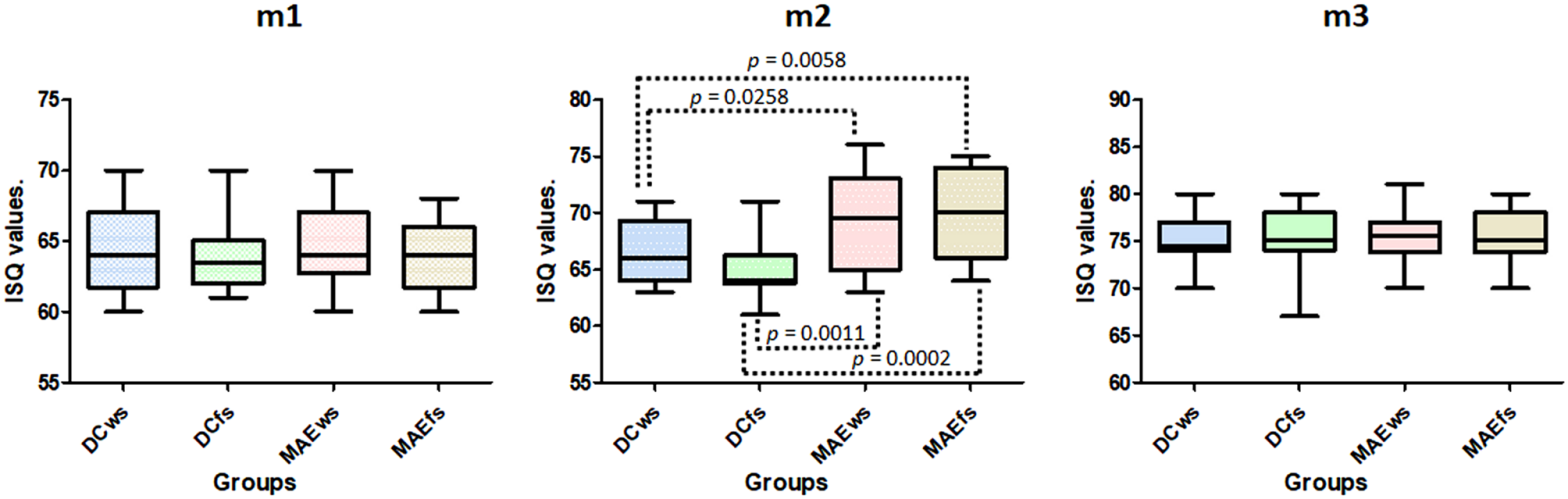
Graphs of data distribution and statistical analysis (*t* test) at the three moments of measurements. Only at moment m2 did the groups show statistical differences.

The results of the ISQ measurements showed a similar value at the first moment (m1) for all groups; no statistical differences were detected (*p* = 0.9070). However, in the second moment (m2) significative differences were observed between the groups (*p* = 0.0002). Whereas, in the last moment (m3), no statistical differences were found between the four groups (*p* = 0.9735). Figure 6 shows the data distribution on the three moments of measurements, and the statistical analysis (*t*-test) comparing the groups that presented differences in the moment m2.

**Figure 7:**
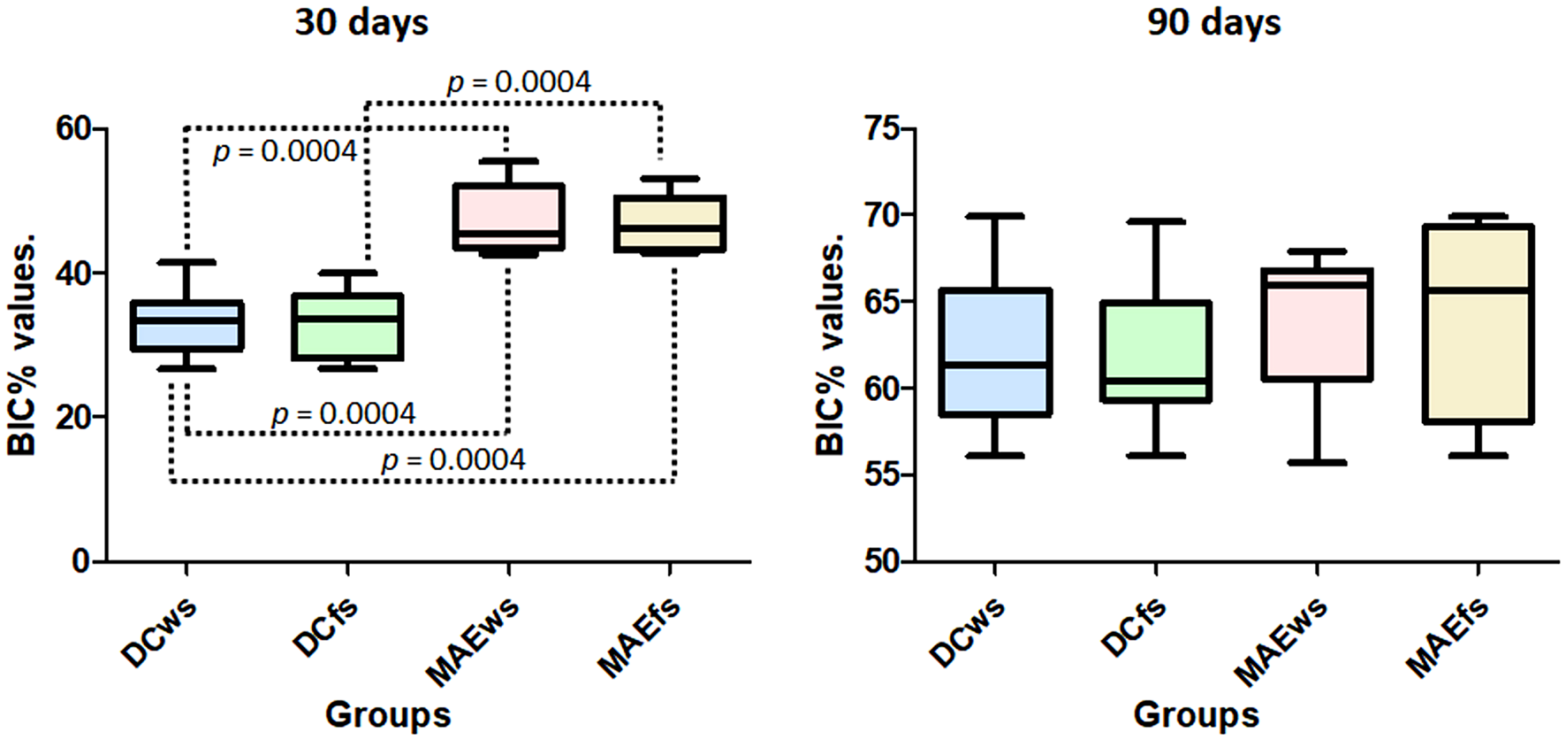
Graphs with the BIC% data distribution and statistical difference obtained in both times for each group.

Regarding the histological evaluations, the BIC% measured in the samples with 30 days of healing from the installation of the implants presented statistical differences between the four groups (*p* < 0.0001). Statistical analysis comparing the groups with each other (Bonferroni test) showed that there was no difference between the groups DCws versus DCfs (*p* = 0.8595) and MAEws versus MAEfs (*p* = 0.9296), whereas the other comparisons between the groups presented differences, which are presented in Fig. 7. However, within 90 days of evaluation, the groups did not show statistical differences (*p* = 0.8440). Figure 7 shows the graph with the data distribution obtained in both times.

**Figure 8:**
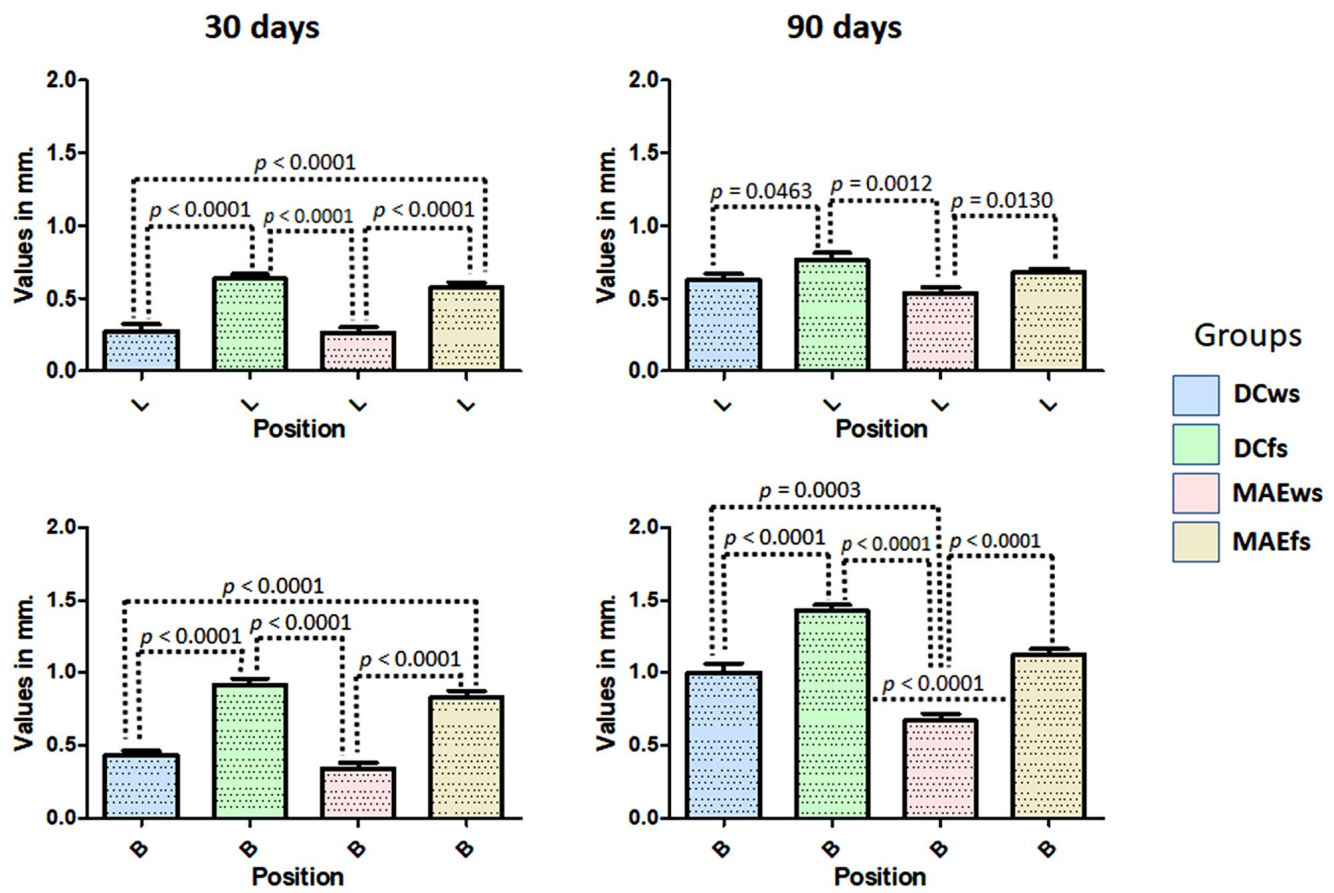
Graphs showing the MBL1 data distribution and the *p*-values between the groups that presented statistically significant differences in both positions (buccal and lingual) and in the two evaluation times (30 and 90 days). *B* buccal and *L* lingual position.

The buccal and lingual (MBL1) evaluations of the samples from each group, different values were verified for both positions, in general, the buccal position showed greater loss in height in all groups. Within 30 days, the samples showed, in both positions (buccal and lingual), a greater loss of bone height for the DCfs and MAEws groups compared to the DCws and MAEws groups, with significant differences between the groups (ANOVA with *p* < 0.0001). At 90 days after the implantations, smaller differences were observed between the groups in the lingual position and greater differences were observed in the buccal position, with the groups where the implants were installed in fresh alveoli (DCfs and MAEfs) had greater loss of bone height compared to the DCws and MAEws groups. When comparing the groups using the Bonferroni test, Fig. 8 shows the data distribution and the *p*-values between the groups that presented statistically significant differences in both positions (buccal and lingual) and in the two evaluation times (30 and 90 days).

**Figure 9:**
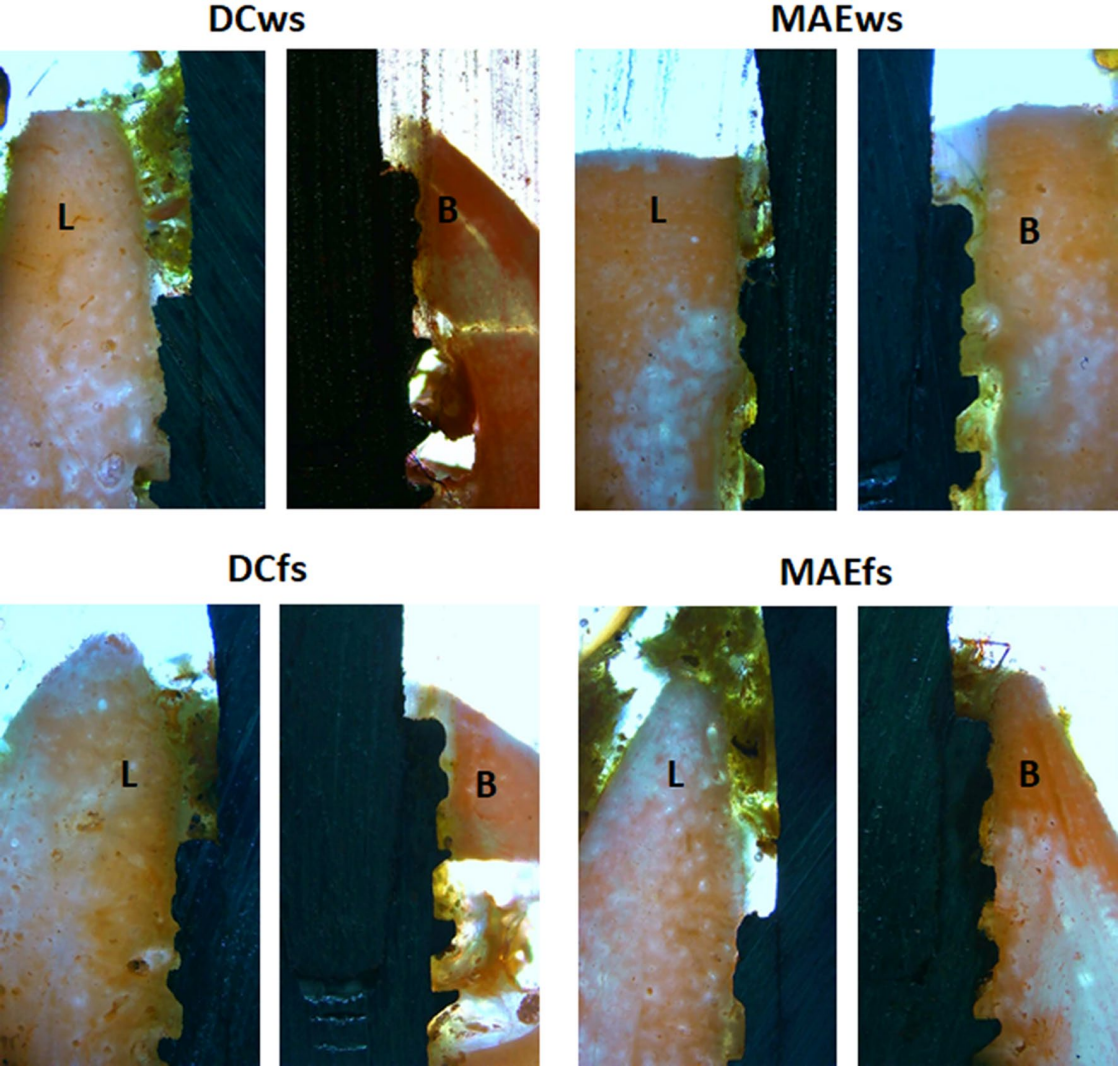
Representative histological images of the buccal (B) and lingual (L) crestal bone behavior of samples for all groups in the time of 30 days. 10 × magnification.

Figure 9 show a representative histological image of the buccal and lingual crestal behavior at the time of 30 days.

**Figure 10:**
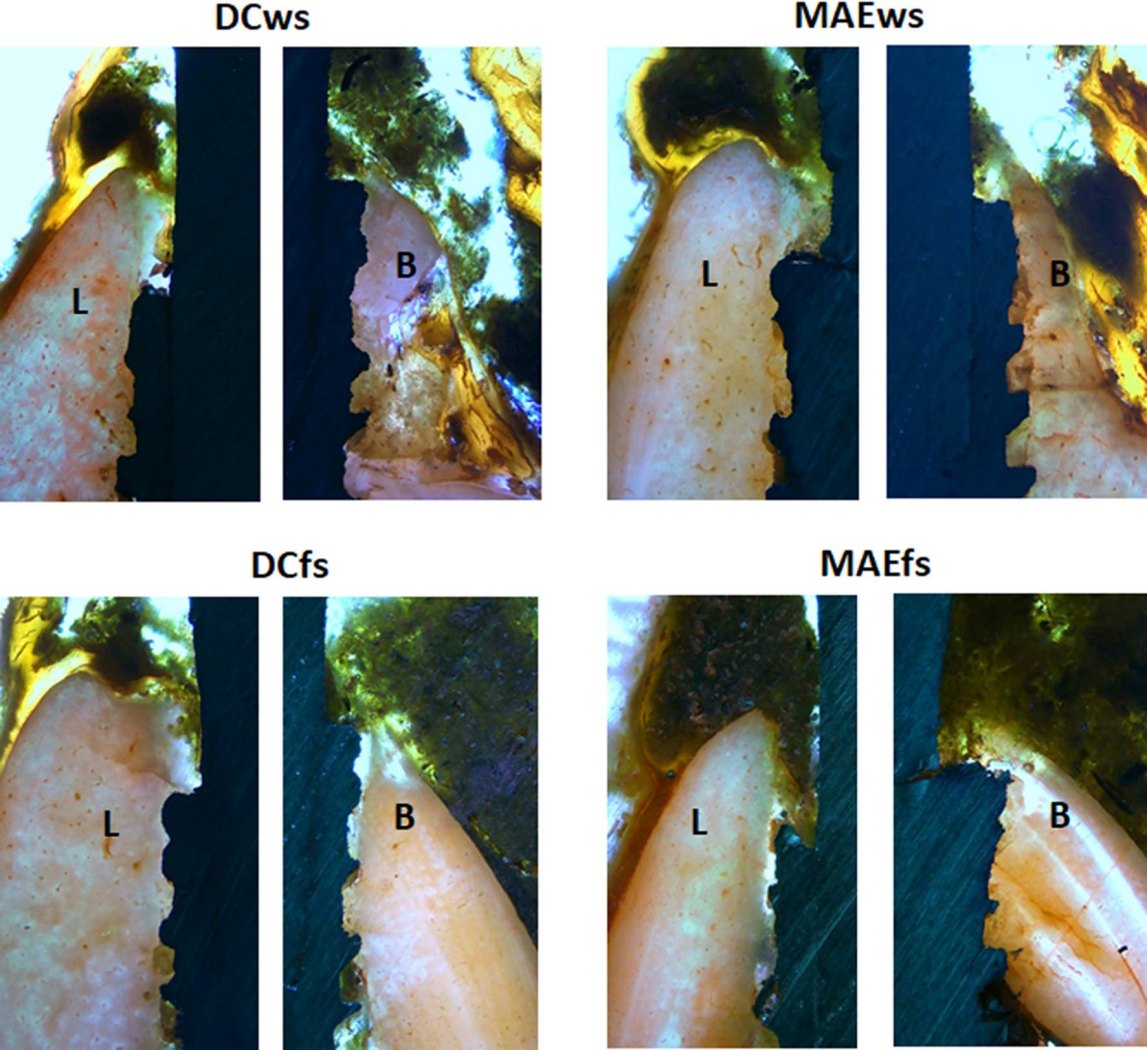
Representative histological images of the buccal (B) and lingual (L) crestal bone behavior of samples for all groups in the time of 90 days. 10 × magnification.

Figure 10 show a representative histological image of the buccal and lingual crestal behavior at the time of 90 days.

Radiographically, the MBL was measured in the medial and distal positions of each implant in relation to the platform of the implants, which were initially positioned 2 mm infra-bone. As the values of each group for the mesial and distal positions were similar, showing no statistical difference in each sample group at both times (*p* < 0.0001), an overall average was taken to analyze the data in each group at each time.

**Figure 11:**
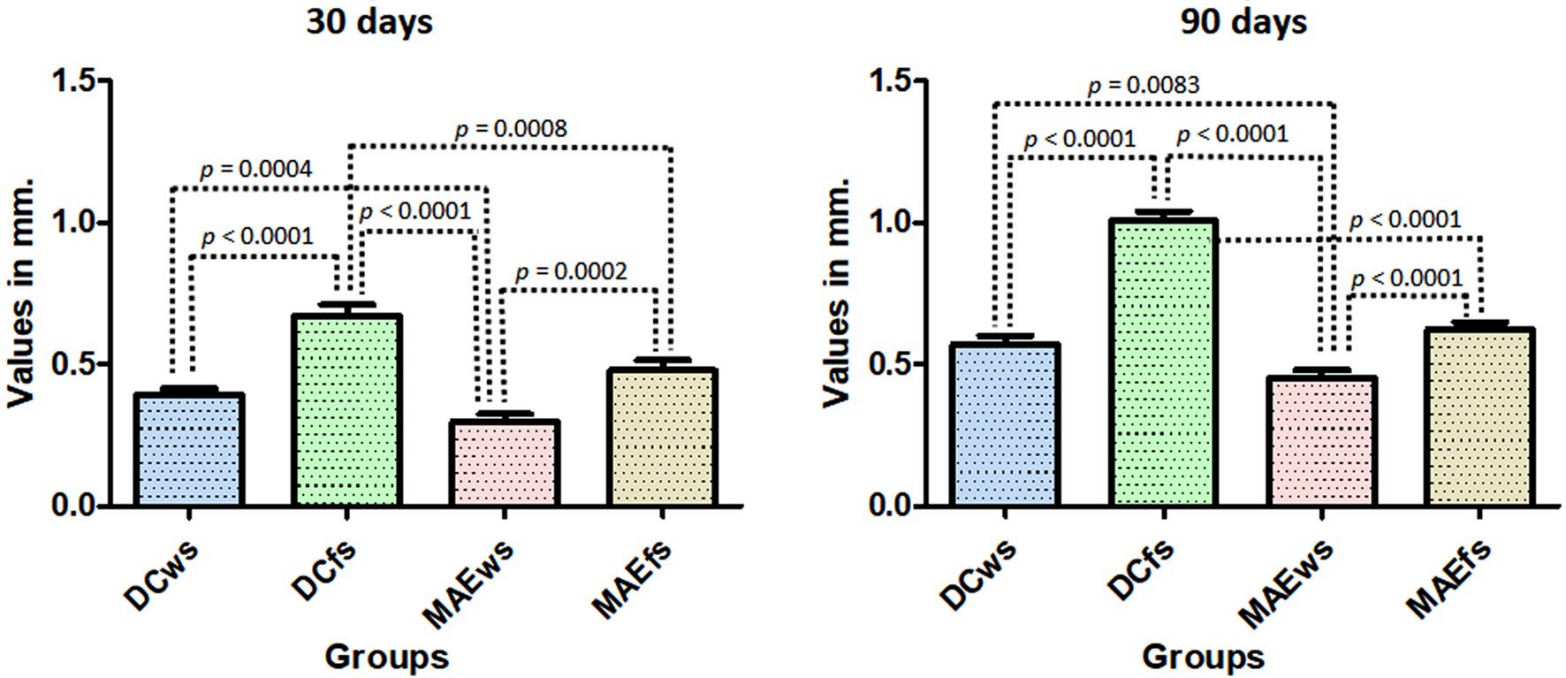
Graph images show the values obtained in each group at the two evaluation times, as well as the *p*-value obtained by the *t*-test between the groups that presented statistical differences.

Figure 11 graphically shows the values obtained in each group in the two evaluation times, as well as the *p*-value obtained by the *t*-test between the groups that presented statistical differences.

**Figure 12:**
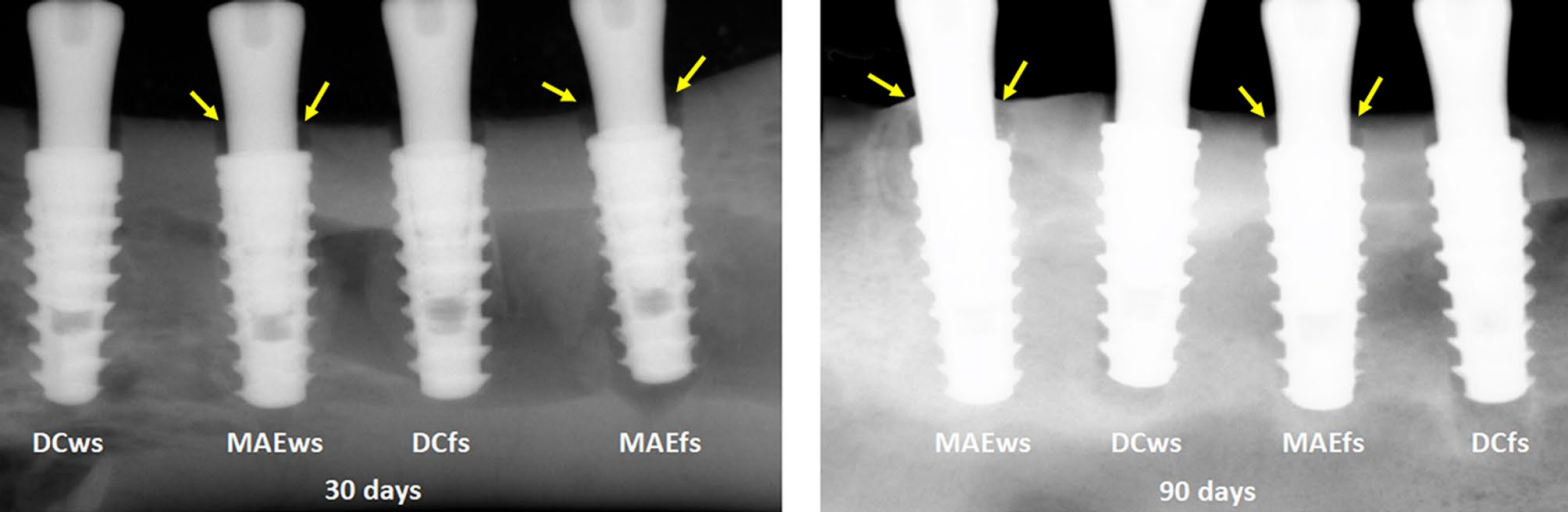
Representative digital X-ray images of each group samples 30 and 90 days after the implantations. Yellow arrows indicating signals of bone formation in the space between the bone and the healing abutment that are more intense in the samples of MAE groups.

Moreover, in Fig. 12, which shows representative radiographic images of samples from each group at 30 and 90 days after implantations, we can observe signs of greater bone formation in the region corresponding to space generated between the bone and the healing abutment in the samples of the Maestro implant groups (MAEws and MAEfs) compared to the DuoCone implant groups (DCws and DCfs).

No positive statistical correlation was found between MBL1 and MBL2 values and implant insertion torque values.

## Discussion

The present experimental in vivo study aimed to evaluate the effects of insertion torque on peri-implant tissues in two models of conical implants with different macrogeometries, one with conventional thread characteristics (Duo Cone implant) and the other with macrogeometry featuring chambers of healing in your body between the threads (Maestro implant), which promote a lower insertion torque to the implants. Both implant models were tested and compared under two distinct conditions, implantation in conventional bone sites (without sockets) and in post-extraction sites (fresh sockets). The results obtained showed that, both in the first assessment time of 30 days and in the proposed second time of 90 days after implantations, the biological processes that led to implant osseointegration and healing of the crestal peri-implant tissues occurred differently at the interface of the implants depending on the macrogeometry and the site condition. The results of the osseointegration process, mainly in relation to the initial evaluation stage (30 days), showed an acceleration of the process in implants that are installed with low torque values, corroborating the results presented by other recently published studies^23,24,27–29^. In the late evaluation period (90 days), no differences were found between the models of implants tested regarding the osseointegration parameters tested (%BIC and ISQ values).

Regardless of the results in implants installed in sites immediately after extraction or in healed sites, several studies showed that, mainly in the buccal bone level, regardless of the technique and implant model used, there will be a greater loss of bone height compared to implants installed in sites without extraction, and it will also be bigger compared to the lingual portion of these implants^10,34–38^. Similar results were obtained in our present study, where the implants of both groups (DCfs and MAEfs) showed greater loss of height in the vestibular portion compared to the groups where the implants were installed in sites without extraction (DCws and MAEws), with a difference of 125% at 30 days and 42.2% after 90 days (in both cases DCfs/MAEfs > DCws/MAEws). While in the mesial and distal portion, the difference between implants installed in fresh alveoli was 76.5% at 30 days and 64.6% after 90 days; in both cases, DCfs/MAEfs > DCws/MAEws.

Directly comparing the two models of implants installed in fresh alveoli, we observed that the implants that were installed with low torque (MAEfs group) had a lower loss of buccal bone crest (− 10% for the 30-day period and − 24% for the 90 days) compared to the DCfs group. While in the mesiodistal portion, the difference was 34% after 30 days and 38% after 90 days, in both cases, MAEfs < DCfs groups. In a clinical study, some authors showed that implants inserted with an insertion torque higher than 50 Ncm showed significantly more bone resorption in comparison with implants with torque less than 50 Ncm^39^. Moreover, in another recent clinical study, in which implants were installed with torque values below 20 Ncm, it was observed that these torque values yielded favorable survival rates with ideal marginal bone levels^25^. These published findings corroborate the results of our present study.

Regarding the behavior of the crestal bone in implants installed in healed sites, the group of researchers led by Professor Barone carried out clinical studies where they evaluated the changes in the crestal bone in implants installed with high insertion torque (≥ 50 Ncm) and regular torque (< 50 Ncm), reporting that implants inserted with high insertion torque (≥ 50 Ncm) showed greater peri-implant bone remodeling and buccal mucosa recession compared to implants inserted with torque < 50 Ncm^40,41^. In our study, the mean insertion torque of the implants in the DC group in the areas of healed sites was 47.2 ± 4.69, very close to the group indicated as regular torque in the previous study, and the MAE implant group presented a mean torque of 29.3 ± 3.16 Ncm, thus being considered a low torque. The results obtained after 30 days showed a 40% lower difference in oral bone loss for the MAE group compared to the DC group, and the difference in mesiodistal bone loss was 44% lower (MAE < DC). While in the evaluation 90 days after implant placement, a 20% difference in the mean values for the buccal portion and 23% in the mesiodistal portion was found, in both cases MAE < DC groups. However, in the lingual portion, no differences were detected between the groups in both evaluation times.

Our results showed a different behavior in the healing process of the crestal bone portion for the two tested implant macrogeometries and for both proposed conditions. As all implant installation procedures were the same for all implants, that is, the osteotomies were performed by the same operator and the same drill sequence was used, the only differences were observed in the insertion torque; the different loss values of the marginal bone were supposedly caused by this factor. Also, regarding the difference in drilling speed, which was different for the 2 implant models studied (following the manufacturer's recommendations), it is important to comment that recent studies have shown that, despite small qualitative and quantitative differences between the pilot holes drilled at low and high speed, these differences were insufficient to cause a statistically significant change in the insertion torque of the implants^42^. Furthermore, other studies have shown that drilling speed does not affect the initial stability parameters of implants^43,44^. However, different results were found in the literature with regards to the healing behavior of peri-implant crestal tissues in relation to insertion torque effects^45–48^. On the other hand, a study by our research group was recently published where surgical trauma during maneuvers for implant placement, in this case bone tissue drilling, can influence the healing response of the bone^49^. Thus, we created the assumption that the insertion torque of the implants can be considered trauma to the peri-implant bone, and this factor may be responsible for the difference in results in favor of the implants in the MAE groups compared to the implants of the DC groups. However, although no statistical correlation was found between the insertion torque and MBL data, the results obtained lead us to accept the hypothesis that the lower insertion torque presented by the implants of the MAEws and MAEfs groups was responsible for the lower crestal bone loss. These findings corroborate previous studies showing that high torque values are responsible for causing crestal bone loss^22,25,26^.

In the present study, all implants were installed 2 mm subcrestal, as the indication of the manufacturer of the implants used was followed and, based on the results obtained, this indication seemed to be a suitable alternative in both types of conditions tested, that is, healed sites and in sites with fresh sockets. Other studies comparing implant placement at the subcrestal level showed that the maneuver should be preferred as it may reduce the probability of the implant becoming exposed in the future and thus avoid the risk of suffering from peri-implant pathologies^50–52^. Furthermore, as the areas that received implants had a thin mucosa, the installation of implants at an infra-osseous level is quite indicated to reduce the loss of bone crests^53^.

Finally, the in vivo experimental model was a sheep mandible because they are very similar to human bone tissue^54^. However, a limitation of this animal study model, which is a ruminant animal, is the type of chewing, where masticatory movements are circumferential; thus, the forces applied to the implants were greater in the lateral direction on the implants.

## Conclusions

Within the limitations of the present study, the findings showed that, although vertical bone remodeling can be observed around all of the implants in both tested sites, the insertion torque is an important factor that should be considered during implant installation. In general, low insertion torque values (Maestro implants) showed better results of MBL when compared to implants installed with higher torque values (Duo Cone implants). Moreover, our results showed that the implants installed in the sites without sockets showed a less MBL in comparison with the implants installed in sites of fresh sockets. Regarding the measured BIC%, implants installed with lower torque values showed better results within 30 days and, after 90 days of healing, no significant differences were found between the groups.
